# Optofluidic Sensing and Sorting of Chiral Drugs Based on Core–Shell Composite Microspheres

**DOI:** 10.3390/s26185852

**Published:** 2026-09-15

**Authors:** Hongze Gao, Wen Yang, Tun Cao

**Affiliations:** School of Optoelectronic Engineering and Instrumentation Science, Dalian University of Technology, Dalian 116024, China; gaohz@dlut.edu.cn (H.G.); yangwen@mail.dlut.edu.cn (W.Y.)

**Keywords:** optofluidic sensing, chiral sorting, core–shell microspheres, chiral drugs

## Abstract

**Highlights:**

A novel core–shell composite sensing microsphere strategy is proposed to carry chiral drugs with small size and chirality, realizing chiral sorting and optofluidic sensing. Furthermore, the structural parameters of the core–shell microspheres are quantitatively optimized to achieve maximum separation velocity, successfully overcoming the background noise induced by inevitable power fluctuations.

**What are the main findings?**

- The shell of the core–shell composite microsphere acts as a carrier to recognition drug chirality, enabling chiral sorting when combined with optofluidic sensing.

**What are the implications of the main findings?**

- The established geometric and hydrodynamic optimization framework provides a theoretical and structural design reference for developing next-generation, label-free, and continuous chiral drug detection platforms.

**Abstract:**

Current all-optical chiral sorting approaches are ineffective for nanoscale drug molecules with weak chirality, as their faint optical response cannot maintain the chiral sorting mechanism. To overcome this physical limitation, the theoretical study proposes a core–shell composite chiral sensing microsphere, in which a drug crystal core is encapsulated by a nematic liquid crystal shell, acting as a chirality-amplifying carrier for optofluidic detection. Using a sodium ibuprofen nanocrystal as a representative weakly chiral drug, the optimal core–shell sensing structure was determined to consist of a nematic liquid crystal E7 shell with a radius of 19 μm and a sodium ibuprofen crystal core with a radius of 9.5 μm. This optimized configuration amplifies the molecular chirality parameter from 10^−6^ to 6.5 × 10^−3^ and yields the maximum transverse separation velocity of 1.19 μm/s. Hydrodynamic calculations demonstrate that when the optimized composite sensing microsphere accumulates an absolute transverse displacement greater than 19 μm within a microfluidic channel, the maximum longitudinal flow velocity of the system reaches 2.4 μm/s, satisfying the geometric threshold required for continuous sensing and sorting. This study provides a design strategy to advance optofluidic sensing and sorting systems for nanoscale chiral drugs.

## 1. Introduction

Chirality refers to the asymmetric property of a three-dimensional spatial structure that cannot be superimposed on its mirror image [1]. Different chiral enantiomers of the same drug often exhibit significant differences in pharmacological efficacy and toxicological characteristics. Therefore, highly sensitive detection and the high-purity sorting of chiral substances are prerequisites for improving therapeutic efficacy and avoiding severe side effects [2]. Conventional chiral analysis and sorting techniques, such as liquid chromatography and preferential crystallization, generally require the introduction of specific chiral selectors [3]. These methods consume large amounts of reagents, readily cause structural damage to target molecules, and are difficult to maintain under long-term continuous operation, thereby limiting their application in high-throughput analysis [4]. The all-optical non-contact manipulation technique based on optomechanical effects exploits the interaction between chiral light and chiral particles to generate chirality-dependent optical forces, providing a new pathway for label-free sorting. For example, vortex beams carrying topological charges can exert handedness-dependent pulling or pushing forces, enabling the size-dependent sorting of single chiral particles [5]. Tightly focused fractional vector beams have been utilized to generate multiple local optical chirality densities for the stable trapping of multiple pairs of enantiomers [6]. Similarly, by tailoring the transverse polarization configuration of an input Gaussian beam, researchers have inversely constructed chirality-sorting optical force pairs to accurately trap opposite enantiomers at distinct predetermined positions [7]. To further enhance sorting throughput at the nanoscale, enantioselective potential wells generated by metasurfaces have been integrated with fluidic drag forces, achieving high-speed continuous sorting of sub-20 nm chiral particles [8]. Beyond translational manipulation, chiral interactions can also induce rotational dynamics, such as the three-dimensional spinning of chiral particles driven by optical intensity-gradient torques [9]. Moreover, the discovery of gradient and curl optical torques has revealed the generation of lateral optical torques, providing new degrees of freedom for rotational optical forces [10]. These cutting-edge mechanisms collectively lay a versatile foundation for developing label-free, non-destructive, and high-throughput continuous optofluidic sensing and sorting platforms.

To overcome the weak optical response of nanoscale substances, researchers have introduced structures such as chiral metasurfaces and plasmonics [8,11,12,13,14,15,16,17], utilizing mechanisms like Fano resonance to significantly enhance local chiral sensing signals. However, these near-field enhancement strategies, similar to conventional optical pulling [18,19,20,21], lateral [22,23,24,25], and gradient forces [26,27,28], are strictly confined to subwavelength regions or localized potential wells, making sustained long-range detection and manipulation in fluidic environments difficult. In contrast, the optical scattering force (ORP) mechanism overcomes spatial confinement and continuously drives particles to undergo macroscopic displacements, serving as an ideal foundation for high-throughput optofluidic sensing platforms. Currently, ORP-based systems typically utilize counter-propagating circularly polarized beams to map the microscopic chiral differences in enantiomers into macroscopic transverse trajectories, which act as visual signals for chiral recognition [29,30,31]. However, when targeting natural nanodrugs with extremely weak chirality, the faint sensing signal is easily completely overwhelmed by achiral background noise from power fluctuations and Brownian motion, leading to failure of conventional optofluidic detection and sorting mechanisms.

To overcome the detection challenges posed by the extremely small size and weak intrinsic chirality of nanodrugs, micrometer-scale auxiliary carriers have been introduced to enlarge the target size and amplify the optical response. Representative strategies include specific capture using liquid crystal microsphere surfaces [32,33,34], chiral mesoporous materials [35,36,37], and chiral metal–organic frameworks (MOFs) [38,39,40]. However, during practical fluidic operation, these interface adsorption mechanisms often struggle to maintain strict enantioselectivity. The occurrence of nonspecific binding tends to introduce interfering sensing signals, thereby compromising final sorting purity.

Using sodium ibuprofen nanocrystals as a representative weakly chiral drug, our study first investigates the physical mechanisms behind the failure of chiral sorting and sensing in counter-propagating circularly polarized optical fields based on the ORP mechanism. To address this issue, a core–shell composite chiral sensing microsphere strategy is proposed, in which a single-chirality drug crystal is encapsulated within a nematic liquid crystal shell. Theoretical analysis indicates that this structure reconstructs the chiral sensing capability by simultaneously amplifying both the target size and intrinsic chirality parameter, while the internal encapsulation circumvents the interference caused by surface nonspecific binding. Furthermore, the geometric dimensions of the core–shell structure are optimized to suppress background noise from achiral scattering forces, and the resulting sensing and separation performance is evaluated through hydrodynamic calculations. This composite sensing carrier provides a theoretical design strategy for mitigating the vulnerability of nanoscale weakly chiral drugs to Brownian motion and background radiation pressure in optofluidic manipulations.

## 2. Optofluidic Sensing Mechanism and Physical Limitations of Nanoscale Chiral Drugs

### 2.1. Counter-Propagating Optical Field Sensing System and Optical Force Model

**Figure 1:**
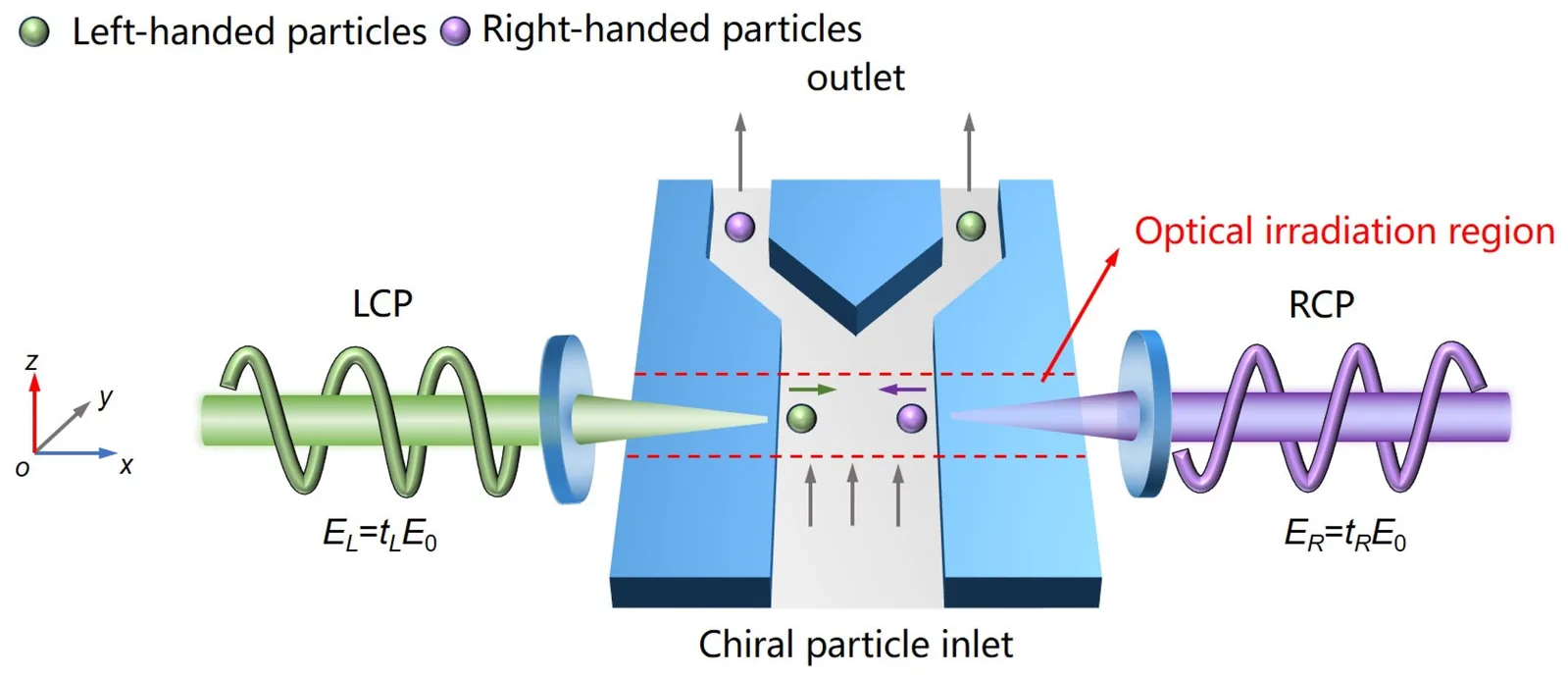
Schematic illustration of the optofluidic sensing system. Chiral enantiomers undergo transverse deflection under the action of counter-propagating LCP and RCP, thereby achieving continuous separation.

The physical configuration of optofluidic chiral sensing and sorting using counter-propagating circularly polarized light is illustrated in Figure 1 [23]. In this configuration, the background solution containing a mixture of chiral enantiomers is injected along the *y*-axis of the microfluidic chip. Within the central optical radiation region, left circularly polarized light (LCP) and right circularly polarized light (RCP) propagate in opposite directions along the *x*-axis, forming the probe field for chiral manipulation. Under the combined action of the longitudinal hydrodynamic drag and the transverse optical force, the chiral particles are deflected toward specific branch channels. This macroscopic transverse displacement essentially serves as the sensing readout signal of the optofluidic system.

In the original scheme described above, the optical powers of the counter-propagating circularly polarized beams must be identical to eliminate the influence of achiral forces on chiral particles. However, in practice, differences among optical components and manual alignment errors inevitably introduce a power difference between the counter-propagating laser beams. By introducing the amplitude transmission coefficients *t_L_* and *t_R_* for the left-side LCP beam and the right-side RCP beam, respectively, the input electric fields ${\vec{E}}_{L}$ and ${\vec{E}}_{R}$ are expressed as

(1) $${\vec{E}}_{L}=t_{L}E_{z}{\vec{e}}_{L}e^{ik_{b}x},{\vec{E}}_{R}=t_{R}E_{z}{\vec{e}}_{R}e^{-ik_{b}x}$$

where both incident lasers are Gaussian beams. *E_z_* is the electric field amplitude under the ideal preset condition and is given by $E_{0}e^{-2z^{2}/w_{0}^{2}}$. Here, *E*_0_ is the electric field amplitude at the center of the Gaussian beam and is expressed as $\sqrt{4P_{0}c\mu_{0}\mu_{b}/\pi w_{0}^{2}n_{b}}$, where *c* is the speed of light, *μ*_0_ is the vacuum permeability, *μ_b_* is the permeability of the background medium, *w*_0_ is the beam waist radius of the Gaussian beam, *n_b_* is the refractive index of the background medium, and *P*_0_ is the initial optical power under the ideal condition. *k_b_* is the wave number in the background medium and is given by 2*πn_b_*/*λ*_0_, where *λ*_0_ is the wavelength of the incident light in vacuum. The variables *x*, *y*, and *z* denote the propagation distances along the *x*-, *y*-, and *z*-axes, respectively. ${\vec{e}}_{L}$ and ${\vec{e}}_{R}$ are the unit polarization vectors of LCP and RCP, respectively, and are expressed as

(2) $${\vec{e}}_{L}=q_{L}\vec{y}-p_{L}\vec{z},{\vec{e}}_{R}=-q_{R}\vec{y}-p_{R}\vec{z}$$

where $\vec{y}$ and $\vec{z}$ are the unit vectors along the *y*- and *z*-axes, respectively. *p_L_* and *q_L_*, together with *p_R_* and *q_R_*, are two pairs of complex numbers, with values of *p_L_* = *p_R_* = 1/√2 and *q_L_* = −*q_R_* = *i*/√2.

The analysis of chiral particles in this study is based on the Tellegen constitutive equation [41], which is expressed as

(3) $$\left\{\begin{array}{l} \vec{D}=\varepsilon_{0}\varepsilon_{s}\vec{E}+i\kappa \sqrt{\varepsilon_{0}\mu_{0}}\vec{H}\\ \vec{B}=-i\kappa \sqrt{\varepsilon_{0}\mu_{0}}\vec{E}+\mu_{0}\mu_{s}\vec{H} \end{array}\right.$$

where *ε*_0_ is the vacuum permittivity, *ε_s_* and *μ_s_* are the relative permittivity and relative permeability of the chiral particle, respectively, and *κ* is the chirality parameter of the chiral particle. A negative value corresponds to left-handed chirality, whereas a positive value corresponds to right-handed chirality.

Based on the analysis of the non-ideal counter-propagating optical field described above, the total transverse optical force *F_x_* acting on a chiral particle under arbitrary power imbalance conditions can be obtained by solving the Maxwell stress tensor integral together with the multipole expansion, yielding

(4) $${\vec{F}}_{x}={\vec{F}}_{non}+{\vec{F}}_{chi}=\sum_{n=1}^{\infty }{\vec{F}}_{non}^{\left(l\right)}+\sum_{n=1}^{\infty }{\vec{F}}_{chi}^{\left(l\right)}$$

where ${\vec{F}}_{non}$ denotes the achiral bias force, which is equivalent to the background radiation pressure, and ${\vec{F}}_{non}$ denotes the chiral force, which drives particles with the corresponding chiral configuration under the action of a single-handed circularly polarized beam. These forces are expressed as

(5) F→nonl=2P0μbλ02π2cw02nbe−4z2/w02ReΓnonltL2−tR2x→F→chil=2iP0μbλ02π2cw02nbe−4z2/w02ReΓchiltL2pLqL∗−pL∗qL−tR2pRqR∗−pR∗qRx→

where $\Gamma_{non}^{\left(l\right)}$ and $\Gamma_{chi}^{\left(l\right)}$ are the *l*th-order scattering factors for the achiral bias force and the chiral force, respectively. Their values are determined by the *l*th-order Mie scattering coefficients *a_l_*, *b_l_*, and *c_l_*. The detailed derivation and complete expressions are provided in Supplementary Section S1.

### 2.2. Influence of Size and Chirality Parameters on Optofluidic Sensing

**Figure 2:**
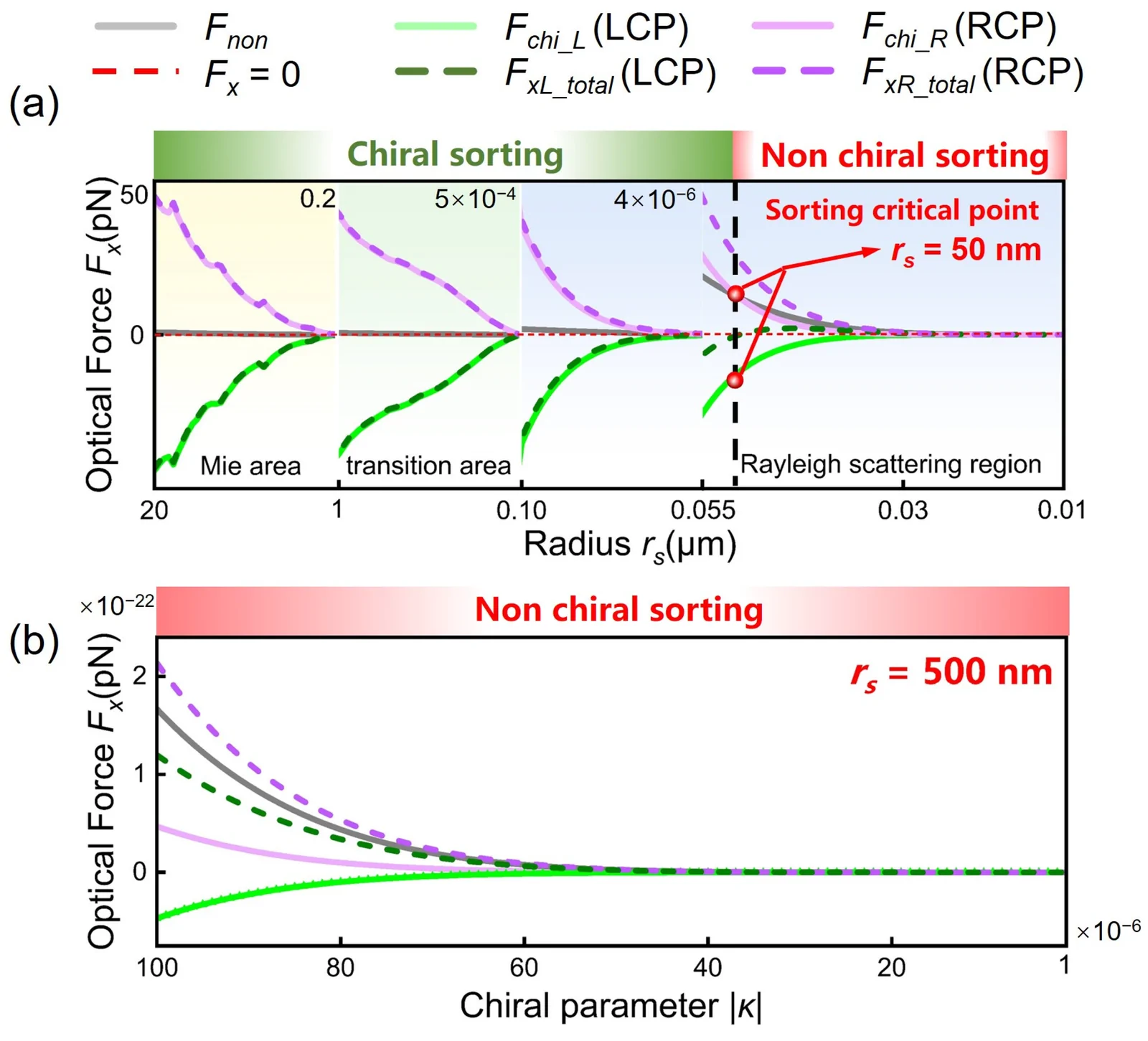
Evolution of the optical forces acting on chiral particles. The initial system parameters are as follows: single-side optical power *P*_0_ = 100 mW; refractive indices of the background medium and the chiral particle are *n_b_* = 1.33 and *n_s_* = 1.6, respectively; particle radius *r_s_* = 20 μm; chirality parameter *κ* = ±0.3; and the ratio of the electric field amplitude transmission coefficients is *t_L_*/*t_R_* = 1.01. *F_non_L_* (LCP) and *F_non_R_* (RCP) denote the achiral optical forces acting on the left-handed and right-handed chiral particles, respectively. *F_chi_L_* (LCP) and *F_chi_R_* (RCP) denote the chiral forces acting on the left-handed and right-handed chiral particles, respectively. *F_xL_total_* (LCP) and *F_xL_total_* (RCP) denote the total transverse optical forces acting on the left-handed and right-handed chiral particles, respectively. Unless otherwise specified, these parameter settings and the corresponding line styles used in the simulations are adopted throughout the remainder of this study. (**a**) Variation in *F_x_* with the chiral particle radius *r_s_*. The red dashed line represents a transverse optical force of zero. *F_non_* denotes the achiral force acting on the chiral particle, and the chirality parameter is *κ* = ±0.3. (**b**) Variation in *F_x_* with the absolute value of the chirality parameter |*κ*| when *r_s_* = 5 μm.

In the practical optical configuration shown in Figure 1, a power imbalance of up to 2% inevitably remains between the two counter-propagating beams, even after precise optical alignment. Assuming that the left optical power is *P_L_* = 100 mW and the right optical power is *P_R_* = 98 mW, the corresponding ratio of the electric field amplitude transmission coefficients is *t_L_*/*t_R_* = 1.01, according to the relationship between optical power and electric field amplitude. During the optical force analysis in this study, the beam waist radius was set to *w*_0_ = 50 μm, the central wavelength of the light source was set to *λ*_0_ = 532 nm, and the chiral enantiomers were assumed to be injected along the flow direction from the transverse center position (*x* = 0 μm). Under these conditions, the evolution of *Fx* as a function of the chiral particle radius *r_s_* and the chirality parameter *κ* is shown in Figure 2.

As illustrated in Figure 2a, when the particle radius *r_s_* gradually decreases and enters the Rayleigh scattering regime, the achiral force *F_non_* decreases more slowly than the chiral force *F_chi_*. Once the particle size falls below a critical threshold, *F_non_* becomes dominant, causing both left-handed and right-handed chiral enantiomers to be driven toward the same side of the microfluidic channel, thereby destroying the optofluidic sensing mechanism. Meanwhile, the intrinsic chirality fundamentally determines the absolute magnitude of *F_chi_*. For weakly chiral materials, the small *F_chi_* is overwhelmed by the *F_non_* induced by even a slight optical power mismatch, as well as by Brownian motion, as shown in Figure 2b. Consequently, both enantiomers are likewise driven toward the same side of the channel.

In summary, the effective sensing and sorting of chiral particles in a non-ideal optical field is jointly governed by the particle size and the chirality parameter. Effective chiral recognition can only be achieved when particles possess both a sufficiently large size and strong chirality. Therefore, for naturally occurring weakly chiral nanodrugs, whose chirality parameter is on the order of $10^{-6}$, a single-dimensional regulation strategy is insufficient. If only the particle size is increased through crystallization, the effective chirality parameter κ remains extremely small, and the unidirectional background radiation pressure still dominates the optical force distribution. Conversely, if only the local optical chirality is enhanced without increasing the nanoscale particle size, the interference caused by background scattering and Brownian motion cannot be overcome. These physical limitations indicate that efficient detection and sorting of weakly chiral drugs in practical systems with optical power fluctuations require a carrier capable of simultaneously enlarging the particle size and amplifying the macroscopic chirality.

## 3. Chirality Amplification and Optofluidic Sensing Based on Core–Shell Composite Sensing Microspheres

### 3.1. Proposed Construction and Theoretical Feasibility Analysis of Core–Shell Composite Chiral Sensing Microspheres

**Figure 3:**
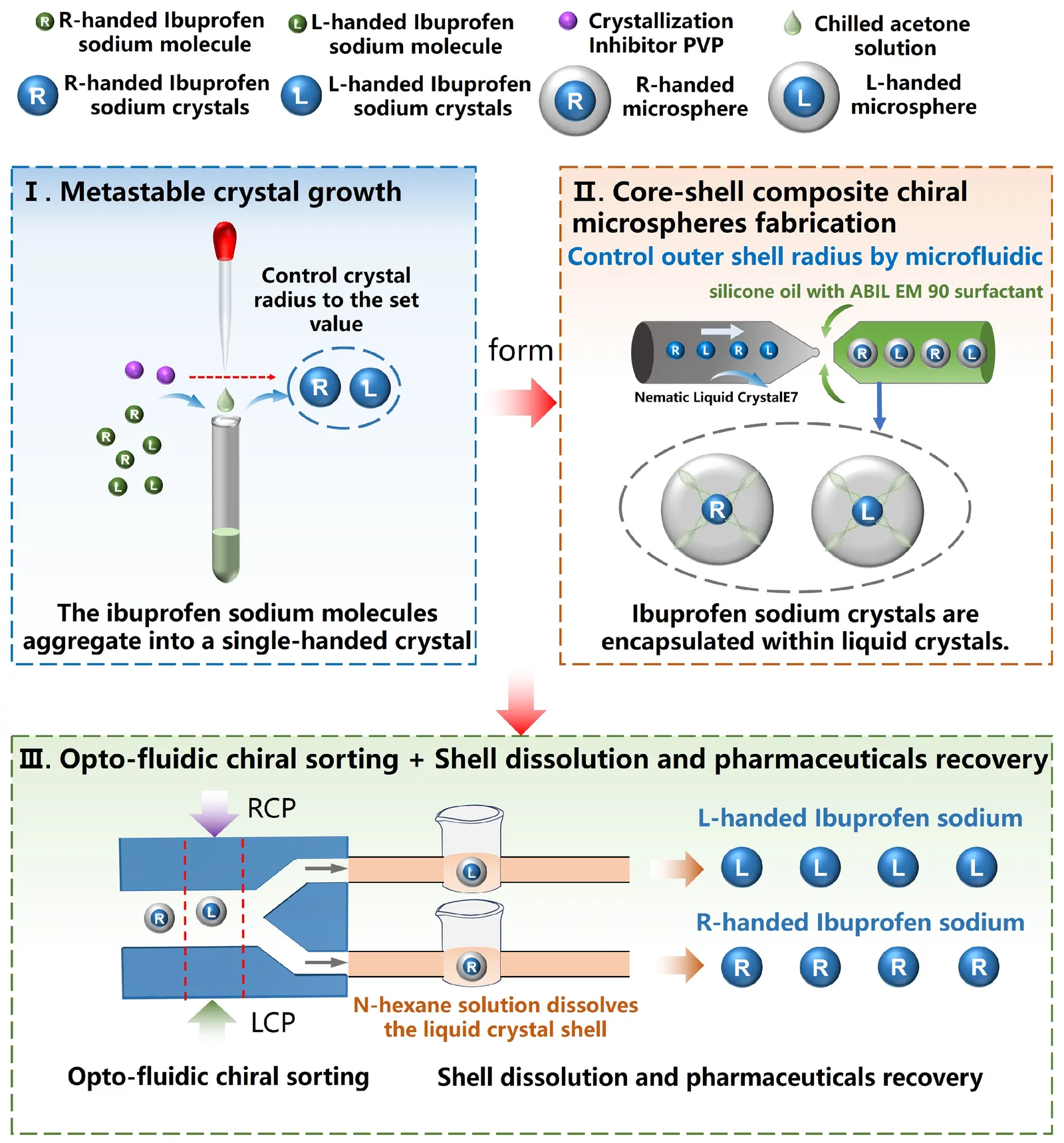
Schematic illustration of the proposed construction, sorting, and recovery process of core–shell composite chiral sensing microspheres. (**I**) Metastable crystal growth stage: Antisolvent crystallization is employed by adding the crystallization inhibitor PVP to an acetone solution containing sodium ibuprofen. By controlling crystallization time and reaction conditions, left- and right-handed sodium ibuprofen molecules are induced to aggregate into single-chirality crystals of predetermined sizes. (**II**) Core–shell composite chiral sensing microsphere preparation stage: A microfluidic technique is used to fabricate liquid crystal microspheres encapsulating sodium ibuprofen crystals. The dispersed phase consists of nematic liquid crystal E7 doped with sodium ibuprofen crystals, while the continuous phase consists of silicone oil with surfactant ABIL EM 90, generating monodisperse core–shell composite structures. (**III**) Optofluidic sensing, sorting, and drug recovery stage: The composite sensing microspheres enter the microfluidic channel, where left- and right-handed microspheres are deflected by optical forces and collected separately. The collected solutions are treated with n-hexane to dissolve the liquid crystal shell, and the separately collected drug-crystal fractions are subsequently recovered by filtration.

To address the physical constraints imposed by weak chirality and small size in chiral drug detection and sorting, this study proposes a core–shell composite chiral sensing microsphere structure, in which a single-chirality drug crystal is encapsulated as the core within a nematic liquid crystal shell. On the one hand, the micrometer-scale nematic liquid crystal shell is proposed to be fabricated via microfluidic droplet techniques, transforming the target from nanoscale drug molecules to the entire composite microsphere and thereby enlarging the geometric size of the object subjected to optical forces. On the other hand, the interface interaction between the drug crystal and the liquid crystal induces forced twisting in the inner liquid crystal layer. Leveraging the long-range orientational coherence of liquid crystals, this distortion propagates outward to form a mesoscopic helical network throughout the micrometer-scale shell, effectively amplifying the faint optical chiral response. Using sodium ibuprofen nanocrystals as a representative weakly chiral drug, the proposed construction route of the core–shell composite sensing microsphere is schematically illustrated in Figure 3.

The construction of the core–shell composite chiral sensing microsphere consists of three primary stages. During the metastable crystal growth stage, an antisolvent crystallization method combined with the crystallization inhibitor polyvinylpyrrolidone (PVP) is employed to induce sodium ibuprofen molecules to aggregate into single-chirality crystals [42,43,44,45]. The low-temperature antisolvent environment suppresses spontaneous racemization of the drug molecules, ensuring the chiral purity of the crystal seeds. Meanwhile, the addition of PVP restricts disordered lattice growth, allowing the crystal size to be controlled at a predetermined scale [46]. During the composite sensing microsphere preparation stage, sodium ibuprofen crystals are stably encapsulated within nematic liquid crystal E7 through shear effects between the continuous and dispersed phases in a microfluidic chip [47,48,49,50]. The surfactant ABIL EM 90 in the continuous silicone oil phase induces planar anchoring of the liquid crystal molecules, maintaining the high monodispersity and stability of the composite microspheres [51,52,53]. Furthermore, this internal physical encapsulation mechanism circumvents common drawbacks of conventional surface-adsorption methods, such as particle desorption and nonspecific binding, thereby enhancing sensing fidelity. During the optofluidic sensing, sorting, and recovery stage, the sorted composite microspheres are collected, and the nematic E7 shell is dissolved using nonpolar n-hexane based on the polarity contrast between the liquid crystal and sodium ibuprofen [54]. Because the sodium ibuprofen crystals remain insoluble in n-hexane [55], the drug-crystal fractions collected from the respective outlet channels can subsequently be recovered through simple physical filtration.

### 3.2. Geometric Optimization and Hydrodynamic Evaluation of Sensing Microspheres

To investigate the optical response of the composite microspheres within a tractable theoretical framework, we adopt an isotropic chiral effective-medium approximation. The average refractive response is represented by $n_{eff}$, while the assumed macroscopic chiral response associated with liquid-crystal twisting is described by *κ_eff_*, as detailed in Supplementary Section S2. This treatment does not explicitly resolve the spatially varying liquid-crystal director or local birefringent anisotropy. Such anisotropy may modify polarization-dependent scattering and resonance positions and strengths, potentially affecting the calculated optical forces, sorting velocities, and optimal dimensions. Spatial integration of the optical force does not, by itself, establish that these effects are negligible. The results presented below should therefore be interpreted as predictions within the adopted effective-medium model. Quantifying the associated error requires comparison with electromagnetic calculations incorporating a specified anisotropic director configuration.

As analyzed in Section 3.1, the effective chirality of the composite microsphere is jointly determined by the sizes of the sodium ibuprofen core and the nematic liquid crystal E7 shell. Assuming the liquid crystal shell radius is *r_out_* and the drug crystal core radius is *r_in_*, the effective chirality parameter *κ_eff_* of the entire sensing microsphere is expressed as

(6) $$\kappa_{eff}=\frac{\lambda_{0}\Phi_{0}}{4\pi \left(r_{out}+K_{22}/W\left(1-\rho \right)\right)}$$

where Φ_0_ is the ideal topological twisting angle of the outer nematic liquid crystal layer induced by the microcrystal surface, *K*_22_ is the twist elastic constant of the liquid crystal bulk, *W* is the azimuthal anchoring strength of the crystal surface, and *ρ* = *r_in_*/*r_out_* is the core–shell radius ratio. For each specific composite microsphere size, the overall refractive index *n_eff_* of the microsphere can be equivalently calculated using the Maxwell–Garnett model. The detailed derivation of the corresponding equation is provided in Supplementary Section S2.

According to Equation (6), $\kappa_{eff}$ increases with a decreasing ρ and an increasing $r_{out}$. However, two physical constraints exist in practical optofluidic applications. First, during microfluidic fabrication, it must be ensured that each liquid crystal droplet encapsulates only a single drug crystal; thus, the minimum value of ρ is set to 0.5. Second, the shell radius $r_{out}$ must be smaller than 60% of the Gaussian beam waist radius $w_{0}$ to guarantee that the microsphere is fully immersed within the core energy region characterized by the most uniform light intensity.

**Figure 4:**
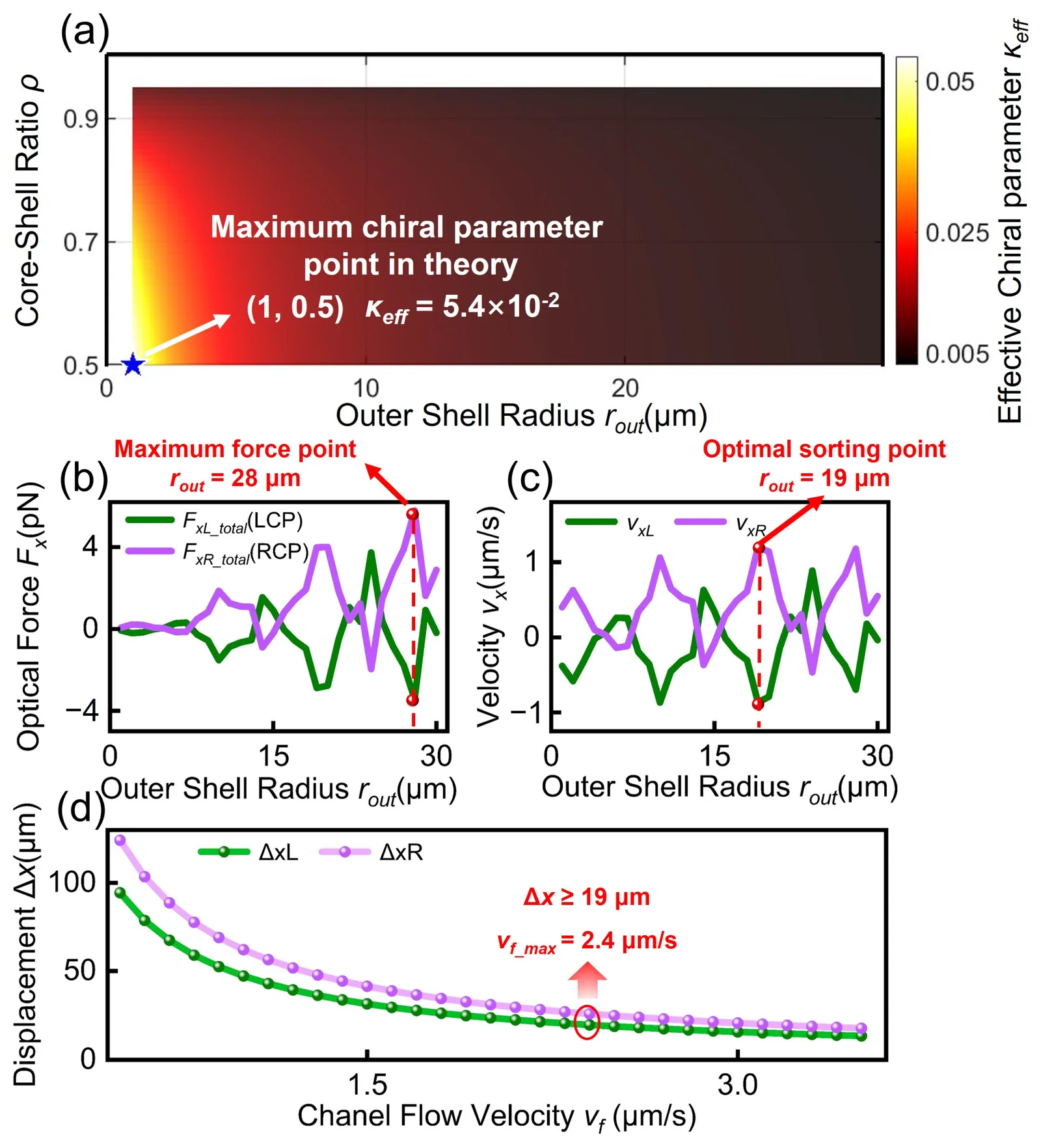
Geometric parameter optimization and optofluidic hydrodynamic evaluation of core–shell composite sensing microspheres. The background solution used for sorting is silicone oil with a kinematic viscosity of 10 cSt and a refractive index of $n_{b}=1.399$. (**a**) Two-dimensional phase diagram showing the evolution of the effective chirality parameter $\kappa_{eff}$ as a function of the core–shell radius ratio ρ and shell radius $r_{out}$. The blue pentagram indicates the theoretical maximum chirality parameter point at (1, 0.5), corresponding to $\kappa_{eff}=5.4\times 10^{-2}$. (**b**) Resonance evolution curves of the transverse optical force acting on the left-handed (solid green line) and right-handed (solid purple line) enantiomer composite microspheres as a function of $r_{out}$ at ρ=0.5. The red dashed line denotes the resonance dimension ($r_{out}=28$ μm) that produces the globally maximum net optical force difference. (**c**) Resonance evolution curves of the transverse terminal velocity $v_{x}$ calculated by incorporating the fluid Stokes drag. (**d**) Absolute transverse displacement Δx accumulated by the optimally configured microsphere under various longitudinal flow velocities $v_{f}$. The red circle indicates the maximum allowable longitudinal flow velocity of $v_{f\_max}=2.4$ μm/s while satisfying the geometric separation threshold of Δx≥19 μm.

Although the theoretical maximum chirality parameter point can be simulated via Equation (6) (indicated by the blue pentagram in Figure 4a), simply pursuing this theoretical maximum is not the global optimal solution for system design. An excessively small particle size sharply reduces the physical scattering cross-section, thereby weakening the optical force acting on the microsphere. Calculations indicate that at $r_{out}=28$ μm, the system reaches a high-order resonance peak, where the left- and right-handed enantiomer microspheres achieve the globally maximum net difference in transverse optical force (Figure 4b).

In practical optofluidic environments, the ultimate physical metric for evaluating chiral separation efficiency is not the static optical force, but the maximum motion velocity $v_{x}$ of the enantiomer microspheres. Under a low-Reynolds-number laminar flow environment, the transverse (*x*-axis) dynamics of the composite microspheres are governed jointly by the total transverse optical force $F_{x}$ and the fluid Stokes drag. The absolute transverse displacement |Δx| accumulated by the microspheres upon exiting the optical field region is expressed as [23]

(7) $$\left|\Delta x\right|=\left|\int_{0}^{t}\frac{F_{x}\left(t\right)}{6\pi \eta r_{out}}dt\right|$$

where η is the fluid viscosity (silicone oil with 10 cSt is used in this study, corresponding to η=9.3 mPa·s). The particle Reynolds number associated with the transverse motion is estimated as $Re_{p}=Ud/\nu$, where U is the particle–fluid relative velocity, $d=2r_{out}\;$ is the microsphere diameter, and ν is the kinematic viscosity of the surrounding fluid. Using U=1.19 μm/s, d=38 μm, and $\nu =10^{-5}\;m^{2}/s$, we obtain $Re_{p}\approx 4.52\times 10^{-6}$, confirming that fluid inertia is negligible at the particle scale.

It should be noted that Equation (7) adopts the unbounded-fluid Stokes drag coefficient $6\pi \eta r_{out}$. Although the microspheres are initially introduced from the transverse center position of the channel, the channel width, height, and particle-to-wall distances are not explicitly specified in the present model. Therefore, possible wall and confinement effects are not quantitatively included. Such effects may increase the hydrodynamic resistance and consequently reduce the predicted transverse velocity and accumulated displacement. The present hydrodynamic results should therefore be interpreted as predictions under the unbounded-fluid approximation.

Under this hydrodynamic approximation, assuming the effective length of the optical radiation region in the main channel is $L_{rad}$ (numerically approximated as $2w_{0}$) and the longitudinal laminar flow velocity of the background fluid is $v_{f}$, the residence time of the microsphere within the optical field is $t=L_{rad}/v_{f}$. Although increasing the shell radius can significantly enhance the optical force via Mie resonance, the resulting viscous drag also increases, suppressing the microsphere velocity. Therefore, the optimal kinetic operating point of the system depends on the maximum net optical force per unit resistance. Following the hydrodynamic conversion that incorporates the fluid viscosity coefficient, the evolution trend of the transverse terminal velocity shifts (Figure 4c). When $r_{out}=19$ μm, the composite microsphere attains a maximum separation velocity of 1.19 μm/s in the fluid. This dimension is identified as the optimal configuration for the microsphere, at which the effective chirality parameter $\kappa_{eff}$ is amplified to $6.5\times 10^{-3}$.

After determining the optimal microsphere configuration, we further evaluate whether the accumulated transverse displacement satisfies the geometric separation criterion under continuous-flow conditions. Within the designated optical radiation region, the longitudinal laminar flow velocity $v_{f}$ determines the effective residence time, which in turn limits the final transverse net displacement Δx upon exiting the field. To ensure reliable capture of the enantiomers by the physical branch channels of the microfluidic Y-junction, the system establishes a geometric separation threshold of Δx≥19 μm. The hydrodynamic evaluation results in Figure 4d demonstrate that, while satisfying this threshold, the maximum allowable longitudinal flow velocity reaches $v_{f\_max}=2.4$ μm/s. This finding theoretically validates the feasibility of the core–shell composite sensing microsphere model for continuous chiral drug sorting under the considered operating conditions.

It should be emphasized that the displacement criterion |Δ*x*| ≥ 19 μm represents a geometric condition for directing the composite microspheres toward different outlet channels, rather than a direct prediction of the enantiomeric purity of the recovered product. In practical operation, the final purity may additionally depend on the distributions of the initial particle positions, microsphere dimensions and effective chirality, Brownian fluctuations, encapsulation uniformity, and microfluidic fabrication tolerances. Since these effects are not statistically incorporated into the present deterministic model, a quantitative product-purity value cannot be reliably obtained from the current calculations.

Table 1 summarizes representative approaches using their respective quantitative metrics. Plasmonic structures can enhance local optical chirality or molecular circular-dichroism readouts [15,17], whereas the chiral MOF films considered here improve enantioselective recognition [40]. In contrast, the present model describes the effective chiral response of a drug-crystal/liquid-crystal composite carrier. Under the assumed model conditions, *κ_eff_* = 6.5 × 10^−3^, corresponding to a nominal ratio of approximately 6.5 × 10^−3^ relative to the weak-chirality reference value of 10^−6^. This ratio characterizes the modeled composite response rather than an experimentally established increase in the intrinsic chirality of individual molecules. Because the reported quantities and reference conditions differ, these values do not provide a common basis for ranking amplification performance across the different platforms.

## 4. Conclusions

The study investigated the optical force mechanisms acting on chiral particles in counter-propagating interference fields and analyzed the physical reasons limiting chiral recognition and sorting as particles evolve from the Mie scattering regime into the Rayleigh scattering regime. The results indicate that under a practical 2% optical power imbalance (*t_L_*/*t_R_* = 1.01), naturally occurring nanoscale weakly chiral drug particles generate extremely faint chiral forces due to their small physical scattering cross-sections and weak intrinsic chirality. Consequently, the specific chiral sensing signal is overwhelmed by the achiral bias force (background radiation noise) and Brownian motion, resulting in the failure of conventional optofluidic chiral recognition and separation.

To address these limitations, a core–shell composite chiral sensing microsphere strategy was proposed, in which a single-chirality drug crystal is encapsulated within a nematic liquid crystal shell. The underlying physical mechanism demonstrates that this structure utilizes surface anchoring induced by the inner crystal to form a mesoscopic helical network throughout the liquid crystal shell, simultaneously amplifying both the geometric size and the effective chirality parameter (from the order of $10^{-6}$ to $6.5\times 10^{-3}$). Furthermore, internal physical encapsulation circumvents the interference caused by surface nonspecific binding.

Based on this composite structure, geometric parameter optimization and hydrodynamic evaluations were performed. Calculation results demonstrate that by balancing the optical force enhancement from Mie resonance against the fluid Stokes drag, the enantiomer microspheres achieve a maximum transverse separation velocity of 1.19 μm/s in fluid when the core–shell radius ratio is 0.5 and the shell radius is optimized to 19 μm. Optofluidic evaluations further confirm that under a maximum longitudinal flow velocity of 2.4 μm/s, the optimized composite microspheres stably accumulate an absolute transverse displacement exceeding 19 μm within the radiation region, satisfying the geometric threshold for microfluidic Y-junction separation.

In summary, this work quantitatively identifies the physical boundaries limiting the detection and sorting of nanoscale chiral drugs in non-ideal optical fields and supports the theoretical feasibility of the core–shell composite sensing microsphere strategy under the considered model conditions. The established geometric optimization and hydrodynamic evaluation framework offers a theoretical foundation and design reference for label-free, and continuous chiral drug sensing, sorting, and subsequent recovery in optofluidic systems.

## Figures and Tables

**Table 1:** Representative strategies for enhancing chiral responses or enantioselective recognition and their respective quantitative metrics.

Strategy	Representative Study	Reported Quantitative Metric	Physical Meaning of the Reported Metric
Plasmonic Fano-resonant metasurface	Cao and Qiu [15]	Maximum local optical chirality-density enhancement *K*/*K_CPL_* of approximately 13 and 16 at the *P*_4_ and *P*_5_ resonances, respectively	Enhances the chirality of the local optical field; the reported quantity is not equivalent to the effective material chirality parameter *κ_eff_* used in this work
Suspended chiral metasurface	Cen et al. [17]	In the analytical comparison, double-sided molecular contact produces a differential circular-dichroism signal Δ*CD* twice that of single-sided contact under otherwise matched conditions	Enhances the chiroptical readout signal rather than the intrinsic or effective material chirality parameter
Macro–microporous chiral MOF film	Yang et al. [40]	Enantioselectivity factor *EF* = 3.28 for M-L-His-ZIF-8 films, compared with *EF* = 1.33 for conventional L-His-ZIF-8 films without macropores	Improves enantioselective recognition; *EF* characterizes the ratio of association constants for two enantiomers and is not directly comparable with
Core–shell drug-crystal/liquid-crystal microsphere	This work	Model-predicted effective chirality parameter |*κ_eff_*| = 6.5 × 10^−3^	Represents the effective chiral response of the composite carrier arising from liquid-crystal reorganization. The resulting $\kappa_{eff}$ characterizes the effective chirality of the entire composite microsphere rather than the intrinsic chirality of the drug molecules.

## Data Availability

The original contributions presented in this study are included in the article. Further inquiries can be directed to the corresponding author.

## Supplementary Materials

The following supporting information can be downloaded at https://www.mdpi.com/article/10.3390/s26185852/s1, Section S1: Derivation of the Optical Force Model; Section S2: Calculation of Effective Chiral Parameter and Refractive Index for the Core–Shell Composite Microsphere Model. References [26,41,56,57,58,59,60,61] are cited in the Supplementary Materials.
