# Supplementary material for: Optofluidic Sensing and Sorting of Chiral Drugs Based on Core–Shell Composite Microspheres
Source: Sensors (Basel). 2026 Sep 15;26(18):5852. doi: 10.3390/s26185852 (PMC13611108; doi:10.3390/s26185852)
Supplement: Supplementary file 1 [file sensors-26-05852-s001.zip › sensors-4520564-supplementary.pdf]

Optofluidic Sorting of Weakly Chiral Drugs Based on a Core-Shell Effective Medium Model

S1. Derivation of the Optical Force Model

The time-averaged optical force $\langle \vec{F} \rangle$ exerted on a chiral particle in a monochromatic electromagnetic field can be rigorously determined by integrating the time-averaged Maxwell stress tensor $\langle \overleftrightarrow{T} \rangle$ over a closed surface enclosing the particle:

$$\langle \vec{F} \rangle = \oint_S \langle \overleftrightarrow{T} \rangle \cdot \vec{n} dS \tag{s1}$$

where $\vec{n}$ denotes the outward unit normal vector to the closed surface. To better understand the physical mechanism, we consider the light scattering process as a superposition of momentum transfers, thereby decomposing the total optical force into two distinct physical components: the interception force $\vec{F}_{int}$ and the recoil force $\vec{F}_{rec}$. Based on Cartesian multipole expansion, the total optical force on the spherical particle is given by the sum of all-order multipole contributions [1]:

$$\vec{F}_x = \sum_{l=1}^{\infty} \vec{F}_{int}^{(l)} + \sum_{l=1}^{\infty} \vec{F}_{rec}^{(l)} \tag{s2}$$

where l represents the truncation order of the multipoles, which depends on the chiral particle radius r_s [2]:

$$l = \begin{cases} x_{\max} + 4x_{\max}^{\frac{1}{3}} + 1\left(0.02 \le x_{\max} \le 8\right) \\ x_{\max} + 4.05x_{\max}^{\frac{1}{3}} + 2\left(8 < x_{\max} < 4200\right) \\ x_{\max} + 4x_{\max}^{\frac{1}{3}} + 2\left(4200 \le x_{\max} \le 20000\right) \end{cases} \tag{s3}$$

where $x_{\max} = \max\{x_0, x_1, x_2\}$, $x_0 = k_b r_s$, $x_1 = k_b((\varepsilon_s\mu_s)^{1/2}+\kappa)r_s$, $x_2 = k_b((\varepsilon_s\mu_s)^{1/2}-\kappa)r_s$, the refractive index of chiral particle $n_s = (\varepsilon_s\mu_s)^{1/2}$.

To further elucidate the mechanism by which the optical force drives particles within the counter-propagating circularly polarized field, the aforementioned optical force, originating from the underlying momentum exchange, is rigorously decoupled into a curl-free conservative gradient force F^g and a divergence-free non-conservative scattering force F^s [1]:

$$\vec{F}_x = \vec{F}^g + \vec{F}^s = \sum_{l=1}^{\infty} \vec{F}_{int}^{(l)g} + \sum_{l=1}^{\infty} \vec{F}_{rec}^{(l)g} + \sum_{l=1}^{\infty} \vec{F}_{int}^{(l)c} + \sum_{l=1}^{\infty} \vec{F}_{rec}^{(l)c} \tag{s4}$$

where

$$
\begin{cases}
\vec{F}_{int}^{(l)g} = (-1)^{l+1} 2\pi(2l+1)\mathrm{Im}[a_l - b_l]\mathrm{Im}\left[Ae^{2ik_b x}\right]\vec{x} \\
\vec{F}_{int}^{(l)c} = 2i\pi(2l+1)\mathrm{Re}[c_l](U_1 - U_2)\vec{x} \\
\vec{F}_{rec}^{(l)g} = (-1)^{l+1} \dfrac{4\pi l(l+2)}{l+1}\left[\vec{F}_{rec,1}^{(l)g} + \vec{F}_{rec,2}^{(l)g}\right] \\
\vec{F}_{rec,1}^{(l)g} = \mathrm{Im}\left[a_l^* a_{l+1} - b_l^* b_{l+1} - \dfrac{2l+1}{l^2(l+2)} a_l b_l^*\right]\mathrm{Im}\left[Ae^{2ik_b x}\right]\vec{x} \\
\vec{F}_{rec,2}^{(l)g} = \mathrm{Re}\Bigg[c_{l+1}\left(a_l^* - b_l^*\right)c_l^*\left(b_{l+1} - a_{l+1}\right) \\
\qquad - \dfrac{2l+1}{l^2(l+2)}\left(c_l b_l^* - a_l c_l^*\right)\Bigg]\mathrm{Im}\left[Xe^{2ik_b x}\right]\vec{x} \\
\vec{F}_{rec}^{(l)c} = -\dfrac{2i\pi l(l+2)}{l+1}\mathrm{Re}\Bigg[c_{l+1}^*\left(a_l + b_l\right) + c_l^*\left(a_{l+1} + b_{l+1}\right) \\
\qquad + \dfrac{2l+1}{l^2(l+2)} c_l^*\left(a_l + b_l\right)\Bigg](U_1 - U_2)\vec{x}
\end{cases}
\tag{s5}
$$

A, X, U_1, and U_2 respectively denotes：

$$
\begin{gathered}
A = q_1 q_2^* - p_1 p_2^*, \quad X = p_1 q_2^* + p_2^* q_1 \\
U_1 = p_1 q_1^* - p_1^* q_1, \; U_2 = p_2 q_2^* - p_2^* q_2
\end{gathered}
\tag{s6}
$$

In Eq. (s5), a_l, b_l, and c_l represent l-th order of Mie scattering coefficients, explicitly formulated as [3]:

$$
\begin{cases}
a_l = \left[A_l^{(2)} V_l^{(1)} + A_l^{(1)} V_l^{(2)}\right] Q_l \\
b_l = \left[B_l^{(1)} W_l^{(2)} + B_l^{(2)} W_l^{(1)}\right] Q_l \\
c_l = \left[A_l^{(1)} W_l^{(2)} - A_l^{(2)} W_l^{(1)}\right] Q_l
\end{cases}
\tag{s7}
$$

where

$$
\begin{aligned}
A_l^{(j)} &= Z_s D_l^{(1)}(x_j) - D_l^{(1)}(x_0) \\
B_l^{(j)} &= D_l^{(1)}(x_j) - Z_s D_l^{(1)}(x_0) \\
W_l^{(j)} &= Z_s D_l^{(1)}(x_j) - D_l^{(3)}(x_0) \\
V_l^{(j)} &= D_l^{(1)}(x_j) - Z_s D_l^{(3)}(x_0) \\
Q_l &= \frac{\psi_l(x_0)/\xi_l(x_0)}{V_l^{(1)} W_l^{(2)} + V_l^{(2)} W_l^{(1)}}
\end{aligned}
\tag{s8}
$$

In Eq. (s8), $\psi_l(x)$ and $\xi_l(x)$ denotes the first order and the third order of Riccati-Bessel function, respectively. Where $D_l^{(1)}(x) = \psi_l'(x)/\psi_l(x)$ and $D_l^{(3)}(x) = \xi_l'(x)/\xi_l(x)$ are the corresponding logarithmic derivatives, $Z_s = \sqrt{\mu_s/\varepsilon_s}$.

By rearranging Eq. (S4) and extracting the chiral and achiral optical force components from

Eq. (S5), the total optical force exerted on the chiral particle within the system is ultimately decomposed into a non-chiral optical force $\vec{F}_{non(m)}^{(l)}$ and a chiral optical force $\vec{F}_{chi(m)}^{(l)}$:

$$\begin{cases} \vec{F}_{non(m)}^{(l)} = \dfrac{2\pi\varepsilon_0 n_b^2 t_m^2 E_z^2}{k_b^2} \mathrm{Re}\left[\Gamma_{non}^{(l)}\right]\vec{x} \\ \vec{F}_{chi(m)}^{(l)} = \dfrac{2i\pi\varepsilon_0 n_b^2 t_m^2 \left(p_m q_m^* - p_m^* q_m\right) E_z^2}{k_b^2} \mathrm{Re}\left[\Gamma_{chi}^{(l)}\right]\vec{x} \end{cases}, \quad m = L, R \tag{s9}$$

Here，L and R represent the left-circularly and right-circularly polarized (LCP and RCP) fields illuminating the system from the left and right sides, respectively, with $\Gamma_{non}^{(l)}$ and $\Gamma_{chi}^{(l)}$ given by:

$$\begin{cases} \Gamma_{non}^{(l)} = \dfrac{(2l+1)}{2}(a_l + b_l) - \dfrac{2l+1}{l(l+1)}\left(a_l b_l^* + c_l c_l^*\right) - \dfrac{l(l+2)}{(l+1)}\left(a_l^* a_{l+1} + b_l^* b_{l+1} + 2c_l^* c_{l+1}\right) \\ \Gamma_{chi}^{(l)} = (2l+1)c_l - \dfrac{l(l+2)}{(l+1)}\left[(a_l + b_l)c_{l+1}^* + (a_{l+1} + b_{l+1})c_l^*\right] - \dfrac{2l+1}{l(l+1)} c_l^* (a_l + b_l) \end{cases} \tag{s10}$$

Therefore, the total optical force acting on the chiral particle in this optical system can be expressed as:

$$\vec{F}_x = \vec{F}_{non} + \vec{F}_{chi} = \sum_{n=1}^{\infty} \vec{F}_{non(L)}^{(l)} + \sum_{n=1}^{\infty} \vec{F}_{non(R)}^{(l)} + \sum_{n=1}^{\infty} \vec{F}_{chi(L)}^{(l)} + \sum_{n=1}^{\infty} \vec{F}_{chi(R)}^{(l)} \tag{s11}$$

After evaluation and algebraic simplification, we arrive at the final results presented in Eqs. (4) and (5) of the main text.

S2. Calculation of Effective Chiral Parameter and Refractive Index for the Core-Shell Composite Microsphere Model

S2.1. Calculation of the Chiral Parameter

In the core-shell composite chiral microsphere, the embedded ibuprofen sodium microcrystal serves as a chiral dopant entity, where its irregular crystalline steps induce a twisted alignment of the liquid crystal molecules, establishing a robust chiral surface anchoring. While the microcrystal surface attempts to induce an ideal topological twist angle Φ_0（set to $\Phi_0 = \pi$ in our model）within the outer nematic liquid crystal shell, the inherent twist elastic energy of the liquid crystal bulk generates a restoring torque to resist this distortion.

Based on the classic Rapini-Papoular surface potential and torque balance model, the actual steady-state topological twist angle Φ_a achieved within the liquid crystal shell is governed by the interplay between the crystal's surface azimuthal anchoring strength W and the bulk twist elastic constant K_{22} of the liquid crystal. For a confined space with an effective shell thickness of $L = r_{out}-r_{in}$, the analytical solution for the macroscopic twist angle is given by:

$$\Phi_a = \frac{\Phi_0}{1 + \dfrac{K_{22}}{W \cdot L}} \tag{s12}$$

For a typical organic single crystal and nematic liquid crystal E7 interface, the anchoring strength is W ≈ 10^{-5} J/m^2[4, 5], with the twist elastic constant of E7 being K_{22} = 7.3 pN [6].

When polarized light passes through the composite microsphere, its polarization plane undergoes an equal rotation alongside the liquid crystal director. Therefore the effective optical rotation θ_{eff} is:

$$\theta_{eff} = \frac{\Phi_a}{D} = \frac{\Phi_a}{2r_{out}} \tag{s13}$$

In terms of Tellegen constitutive relations, the relationship between κ and effective optical rotation θ_{eff} is:

$$\kappa = \frac{\lambda_0 \theta_{eff}}{2\pi} \tag{s14}$$

By substituting Eq. (S13) into Eq. (S14), we finally obtain the overall conversion equation for the effective chiral parameter κ_{eff} which accounts for the surface anchoring effect:

$$\kappa_{eff} = \frac{\lambda_0}{4\pi r_{out}} \left(\frac{\Phi_0}{1 + K_{22}/W\left(r_{out} - r_{in}\right)} \right) \tag{s15}$$

We assume that $\rho = r_{in}/r_{out}$, which represents the ratio of the core radius to the shell radius. Thus, Eq. (S15) can be rewritten as:

$$\kappa_{eff} = \frac{\lambda_0 \Phi_0}{4\pi\left(r_{out} + K_{22}/W\left(1-\rho\right)\right)} \tag{s16}$$

S2.2. Calculation of effective refractive index

The average refractive index of the liquid crystal $\overline{n}_{out}$ is determined by its ordinary refractive index n_o and extraordinary refractive index n_e, given by [7]:

$$\overline{n}_{out} = \sqrt{\frac{\left(2n_o^2 + n_e^2\right)}{3}} \tag{s17}$$

By treating the core-shell composite chiral microsphere as a unified entity, the effective refractive index n_{eff} can be evaluated using the Maxwell-Garnett model [8]:

$$n_{eff} = \overline{n}_{out} \sqrt{\frac{n_{in}^2 + 2\overline{n}_{out}^2 + 2f\left(n_{in}^2 - \overline{n}_{out}^2\right)}{n_{in}^2 + 2\overline{n}_{out}^2 - f\left(n_{in}^2 - \overline{n}_{out}^2\right)}} \tag{s18}$$

where n_{in} denotes the average refractive index of the homochiral ibuprofen sodium crystal core, f represents the volume fraction of the crystal within the liquid crystal microsphere, which is determined by Eq. (S19):

$$f = \left(\frac{r_{in}}{r_{out}}\right)^3 \tag{s19}$$

Here the ordinary and extraordinary refractive indices of the liquid crystal E7 are n_o = 1.523 and n_e = 1.653, respectively. while the mean refractive index of the homochiral ibuprofen sodium

crystal core is $n_{in} = 1.55$.

References

1. Zheng, H.X.; Li, X.; Ng, J.; Lin, Z.F. Tailoring the Gradient and Scattering Forces for Longitudinal Sorting of Generic-Size Chiral Particles. *Opt. Lett.* **2020**, *45*, 4515–4518.

2. Wiscombe, W.J. Improved Mie Scattering Algorithms. *Appl. Opt.* **1980**, *19*, 1505–1509.

3. Zheng, H.X.; Chen, H.J.; Ng, J.; Lin, Z.F. Optical Gradient Force in the Absence of Light Intensity Gradient. *Phys. Rev. B* **2021**, *103*, 035103.

4. Kumar, P.; Oh, S.Y.; Baliyan, V.K.; Dey, S.K.; Kang, S.-W.; Lee, S.H. Topographically Induced Homeotropic Alignment of Liquid Crystals on Self-Assembled Opal Crystals. *Opt. Express* **2018**, *26*, 8385–8396.

5. Smalyukh, I.I. Liquid Crystal Colloids. *Annu. Rev. Condens. Matter Phys.* **2018**, *9*, 207–226.

6. Donisi, D.; Asquini, R.; d'Alessandro, A.; Bellini, B.; Beccherelli, R.; De Sio, L.; Umeton, C. Distributed Feedback Grating in Liquid Crystal Waveguide: A Novel Approach. *Opt. Express* **2009**, *17*, 5251–5256.

7. Nagaraj, M.; Görtz, V.; Goodby, J.W.; Gleeson, H.F. Electrically Tunable Refractive Index in the Dark Conglomerate Phase of a Bent-Core Liquid Crystal. *Appl. Phys. Lett.* **2014**, *104*, 021903.

8. Hosein, I.D.; Liddell, C.M. Homogeneous, Core-Shell, and Hollow-Shell ZnS Colloid-Based Photonic Crystals. *Langmuir* **2007**, *23*, 2892–2897.
